# Detection of Viral RNA in Tissues following Plasma Clearance from an Ebola Virus Infected Patient

**DOI:** 10.1371/journal.ppat.1006065

**Published:** 2017-01-05

**Authors:** Mirella Biava, Claudia Caglioti, Licia Bordi, Concetta Castilletti, Francesca Colavita, Serena Quartu, Emanuele Nicastri, Francesco Nicola Lauria, Nicola Petrosillo, Simone Lanini, Thomas Hoenen, Gary Kobinger, Alimuddin Zumla, Antonino Di Caro, Giuseppe Ippolito, Maria Rosaria Capobianchi, Eleonora Lalle

**Affiliations:** 1 National Institute for Infectious Diseases “Lazzaro Spallanzani” IRCCS, Via Portuense, Rome, Italy; 2 International Public Health Crisis Group (IPHCG); 3 Institute of Molecular Virology and Cell Biology, Friedrich-Loeffler-Institut, Federal Research Institute for Animal HealthInsel Riems, Germany; 4 Research Centre on Infectious Diseases, Faculty of Medicine, Université Laval, Québec Canada; 5 University College London and NIHR Biomedical Research Centre, University College London Hospitals NHS Foundation Trust, London, United Kingdom; Thomas Jefferson University, UNITED STATES

## Abstract

An unprecedented Ebola virus (EBOV) epidemic occurred in 2013–2016 in West Africa. Over this time the epidemic exponentially grew and moved to Europe and North America, with several imported cases and many Health Care Workers (HCW) infected. Better understanding of EBOV infection patterns in different body compartments is mandatory to develop new countermeasures, as well as to fully comprehend the pathways of human-to-human transmission. We have longitudinally explored the persistence of EBOV-specific negative sense genomic RNA (neg-RNA) and the presence of positive sense RNA (pos-RNA), including both replication intermediate (antigenomic-RNA) and messenger RNA (mRNA) molecules, in the upper and lower respiratory tract, as compared to plasma, in a HCW infected with EBOV in Sierra Leone, who was hospitalized in the high isolation facility of the National Institute for Infectious Diseases “Lazzaro Spallanzani” (INMI), Rome, Italy. We observed persistence of pos-RNA and neg-RNAs in longitudinally collected specimens of the lower respiratory tract, even after viral clearance from plasma, suggesting possible local replication. The purpose of the present study is to enhance the knowledge on the biological features of EBOV that can contribute to the human-to-human transmissibility and to develop effective intervention strategies. However, further investigation is needed in order to better understand the clinical meaning of viral replication and shedding in the respiratory tract.

## Author Summary

An unprecedented Ebola outbreak occurred in 2013–2016 in West Africa. In order to better understand EBOV infection patterns in different body compartments, we have longitudinally explored the presence of already assessed markers of ongoing EBOV replication (negative sense genomic RNA and positive sense RNA) in the upper and lower respiratory tract, as compared to plasma and other body compartments, in a Health Care Worker infected with EBOV in Sierra Leone, who was hospitalized in the high isolation facility of the National Institute for Infectious Diseases “Lazzaro Spallanzani” (INMI), Rome, Italy. The presence of total EBOV RNA and replication markers was observed in specimens of the lower respiratory tract, even after viral clearance from plasma, suggesting possible local replication. Our results contribute to the knowledge on the biological features of EBOV and shed light on the potential role of respiratory compartment in human-to-human transmissibility.

## Introduction

The Ebola virus (EBOV) Makona variant is a negative-sense RNA virus and a member of the Filoviridae. From December 2013 to January 2016, this EBOV Makona caused the largest viral hemorrhagic fever epidemic ever reported in West Africa. [1] Several patients received care in Europe and North America. [2] Among those, two were medically evacuated to the National Institute for Infectious Diseases (INMI) “Lazzaro Spallanzani” of Rome, Italy. The clinical findings of the first imported case to Italy confirmed for Ebola Virus Disease (EVD) have been previously reported [3,4]. Like other patients, who received care outside of Africa, this patient presented a significant lung involvement, consistent with interstitial pneumonia, suggesting that EBOV could cause lung damage, perhaps through replication in these tissues. [4–6] The second imported case to Italy was a Health Care Worker (HCW) working in an Ebola Treatment Center (ETC) in Sierra Leone. He was admitted to INMI for EVD on May 13, 2015 and was discharged in good clinical condition on June 10, 2015. Oral favipiravir (Toyama Chemical Co, Japan), monoclonal antibodies against EBOV (Mil77, Mabworks Beijing China) and sustained hydration were administered, followed by progressive clinical improvement. A longitudinal characterization of cellular immune response has been described in detail elsewhere [7]. At admission, on day three from onset of symptoms, the patient had fever (39.0°C), cough, and a delayed febrile thrombocytopenic syndrome with pericardial effusion; chest X ray showed a basal mild interstitial lung involvement with 89% oxygen saturation. There is no direct evidence suggesting that EBOV can infect lung tissue in humans, although the potential role of EBOV infection of respiratory system has been suggested by clinical in vitro experiments, animal studies and clinical case reports [4,5,8–12]. In addition, viral inclusions can be found inside alveolar macrophages, and free viral particles can be seen within alveolar spaces. In this study, we compared the levels of EBOV negative sense genomic RNA (neg-RNA) and positive sense RNA (pos-RNA), including both mRNAs and anti-genomic RNAs, as indicators of viral replication in clinical specimen of the second EVD patient treated at INMI [13,14], to explore the potential role of different body compartment in EBOV replication.

## Results

### Plasma and sputum clinical specimens

**Fig 1:**
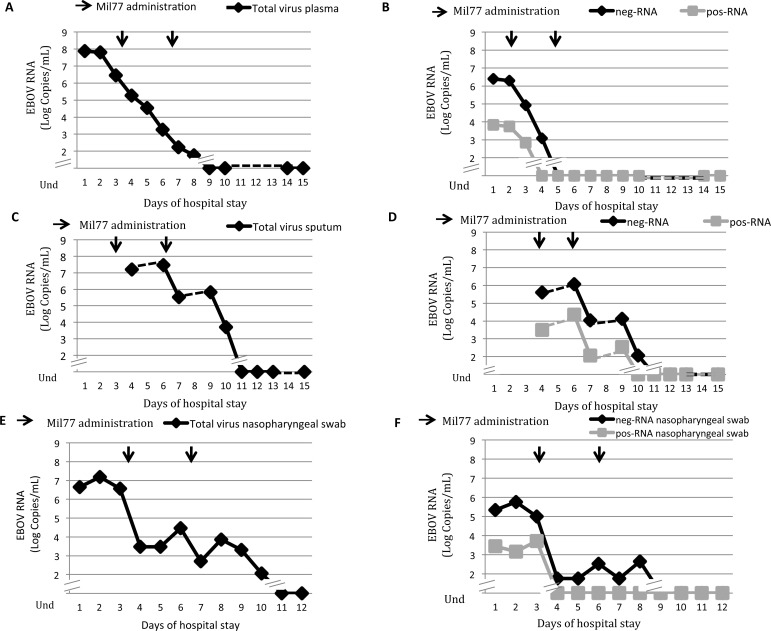
EBOV RNA trends in plasma, sputum and nasopharyngeal specimens during the first 15 days of hospitalization of the second Italian Ebola patient attending the National Institute for Infectious Diseases in Roma (INMI2). Clinical specimens from the patient were collected daily (plasma and nasopharyngeal swab) and regularly throughout hospitalization (Sputum). Arrows indicate the administration of the experimental drug Mil77 (day 3 and day 6). Dotted lines represent the hypothetical trend of those samples not available for this study. Panel A: trend of total EBOV RNA (viremia), which becomes undetectable at day 9. Panel B: trends of neg-RNA and pos-RNA in plasma, which become undetectable at day 5 and 4, respectively. Panel C: trend of total virus RNA in sputum, which becomes undetectable at day 11. Panel D: trends of neg-RNA and pos-RNA in sputum, which become undetectable at day 11 and 10, respectively. Panel E: trend of total EBOV RNA in nasopharyngeal swab. In nasopharyngeal swab the total EBOV RNA reaches undetectable levels at day 13 of hospitalization. Panel F: trends of neg-RNA and pos-RNA in nasopharyngeal swab, which become undetectable at day 10 and 6, respectively. Symbols are specified in the panels.

Fig 1 (Panels A and C) shows the trends of total viral RNA in plasma (viremia) and in sputum. High EBOV viremia was detected in the first days of hospital admission (with a peak of 10^8^ copies/mL at day 1) with a marked decrease at day 3, also overlapping with the administration of Mil77. Declining EBOV viremia continued after further administration of MIL77 on day 6, and reached undetectable levels on day 9. In contrast, total EBOV RNA in sputum peaked at day 6 with above 10^7^ copies/mL and remained high at around 10^6^ copies/mL up to day 9 before becoming undetectable at day 11. Fig 1 shows neg-RNA and pos-RNA levels, in plasma (Panel B) and in sputum (Panel D). High levels of neg-RNA and pos-RNA, were detected in plasma up to day 4 and 3, respectively. In the respiratory compartment, neg-RNA was present at a concentration of 10^6^ copies/mL up to day 11, and pos-RNA was present at a concentration of 10^4^ copies/mL up to day 10 (Panel D). The levels of neg-RNA and pos-mRNA in sputum samples were 2 or 3-Log higher than neg-RNA and pos-RNA values in plasma, respectively.

### Total, neg- and pos-RNA in other clinical specimens

The presence of total, neg- and pos-RNA in nasopharyngeal swab is shown in Fig 1. As depicted, total RNA was present at high levels (10^6^ copies/mL) until day 5, and detected at low levels (10^3^ copies/mL) until day 12 before becoming undetectable at day 13 (Panel E). The prolonged detection of total RNA was paralleled by that of neg-RNA, which was detectable (10^2^−10^3^ copies/mL) until day 10 (Panel B) while pos-RNA became undetectable on day 6 (Panel F). In ocular swabs and urine samples, total RNA remained detectable up to day 15 (10^3^ copies/mL) and day 4 (10^4^ copies/mL), respectively. Interestingly, pos-RNA was always undetectable in all specimens analyzed from these compartments, whereas neg-RNA was detectable around 10^2^ copies/mL both in urine and ocular swab.

## Discussion

This study presents novel insights in the potential role of the respiratory compartment in EBOV replication in a human case of EVD. Several clinical findings suggest lung involvement in different cases of EVD who received care in Europe [4] and the USA [5]. The chest radiography performed in patients treated in the USA revealed bilateral pulmonary infiltrates suggestive of pneumonia [5]. Moreover, the first EVD patient treated in Italy developed significant lung injury, consistent with interstitial pneumonia [4]. The role of neg-RNA and pos-RNA levels as surrogate markers of ongoing EBOV replication has already been established in *in vitro* experiments [13]. We confirmed the specificity of this method using VeroE6 infected cells, where at 24 hours post-infection we observed that the majority of pos-RNA (62%) was cell-associated, while neg-RNA was mostly (84%) shed in the supernatant. In addition, the sensitivity and the linearity of the test were unaffected by sample matrix composition, since plasma and sputum spiked with EBOV viral RNA (Ct 22,12 and Ct 22,41, respectively, for the RNA concentration 10^5^) did not influence tests results as compared to tissue culture medium. Further, no appreciable background noise signal was observed in unspiked plasma and sputum matrices (Ct > 45). Hence, we longitudinally explored the persistence of viral replication markers in clinical samples from the upper and lower respiratory tract of the second EVD patient in Italy, and made comparison with plasma samples. The results indicate the decay of EBOV total RNA, neg-RNA and pos-RNA overtime, which was significantly different in plasma when compared to respiratory samples. In particular, we observed a persistence of total viral RNA and replication markers in sputum, until the second week of hospital stay (up to day 10), when viremia was already undetectable. From these data the possibility that the prolonged detection of EBOV RNA in sputum merely reflects enhanced stability of EBOV RNA in this compartment cannot be ruled out [16]; nevertheless the detection of pos-RNA together with neg-RNA in sputum (until day 9 and 10 of hospital stay, respectively) supports the concept of active viral replication within the respiratory tract, rather than plasma spill-over or prolonged RNA stability. Indeed, the detection of the replication markers in respiratory samples combined with the absence of detectable neg-RNA in the plasma from day 5, while neg-RNA could still be detected for days in respiratory samples, can be explained by active replication of EBOV in lung tissues. Moreover, the absence of detectable levels of pos-RNA in nasopharyngeal swabs (starting day 6 and onward) suggests that the presence of pos-RNA in sputum is likely due to replicating EBOV in the lower rather than in the upper respiratory tract. Additional observations are indicative of a direct role of EBOV replication in lung injury in animals (including nonhuman primates) and in post-mortem examination of human that succumbed with EVD [12]. Moreover, a recent study evaluated the pathogenicity in rhesus macaques of 2 different isolates of the EBOV Makona strains compared to the EBOV Mayinga isolate, showing higher lung pathology in macaques infected with the two Makona strains [17]. In pigs, EBOV can replicate to high titers in the lower respiratory tract and cause severe lung diseases following mucosal exposure [18]. Guinea pigs challenged with aerosolized EBOV can develop lethal interstitial pneumonia [8] while rhesus monkeys demonstrate a significant respiratory involvement [9] with cell-associated EBOV virus antigens present in airway epithelium, alveolar pneumocytes, and macrophages [10,11]. The hypothesis that EBOV can actually replicate into human airway is supported by a recently published review, which collected data from post mortem examinations of 89 fatal cases of filoviral infections (24 of which with EBOV) [12]. This analysis provides evidence of viral replication within the respiratory tract, showing the presence of viral antigens in several lung tissues, with EBOV nucleic acids detected in resident macrophages [12]. Unfortunately, we were only able to recover replication competent virus from plasma collected at presentation and were unable to recover replication competent virus from later plasma samples or other clinical specimens, despite numerous attempts in VeroE6 cell culture (especially from sputum). Since other Authors report difficulties in EBOV isolation from non-blood samples, it is possible that the actual sensitivity of cell culture isolation is not sufficient to rule out virus viability and infectivity [19–20]. To this respect, several factors, among which suboptimal sensitivity of the virus culture system, storage at -80°C and presence of antibodies against EBOV in the clinical samples, may have contributed to unsuccessful virus culture attempts. Despite the lack of supportive data, a key additional question is whether primary pulmonary infection and respiratory transmission of EBOV could actually occur [6]. The presence of virus replication markers here reported adds some support to this possibility, although the evidence is far from being conclusive, due to the absence of recovery of infectious virus in respiratory secretion. Further investigation is needed to better understand the clinical significance of the involvement of the respiratory tract in viral replication and shedding, and possible contribution to respiratory impairment and human–to–human transmission.

## Materials and Methods

Patient’s clinical specimens were longitudinally collected for diagnostic purposes. Plasma samples, nasopharyngeal swabs, ocular swabs and urine samples were collected daily (starting from day 1, day 3, day 1 and day 1, respectively) while sputum samples (mucus and phlegm coughed-up from the lower airways) were repeatedly collected (starting from day 4) throughout hospitalization. We made all efforts to uniform the time (morning) and size (4 mL) of sample collection, in order to minimize this as possible source of difference. Inactivation of samples was performed in the BSL4 facility and RNA extraction was carried out in the BSL2 laboratory. RNA extraction was performed with QIAamp Viral RNA Mini Kit (Qiagen) following manufacturer’s instructions. Identical volumes (140 μl) of each sample have been used for RNA extraction. The quality of all the samples was checked using a house-keeping gene (RNaseP) in qRT-PCR according to [15]. Total virus-specific RNA (total RNA) was measured using quantitative Real-time PCR, RealStar Filovirus Screen RT-PCR Kit 1.0 (Altona Diagnostics GmbH), targeting L gene. To measure EBOV-specific negative sense genomic RNA (neg-RNA) and positive sense RNA (pos-RNA) [including both replication intermediate (cRNA) and messenger RNA (mRNA)], L-gene specific reverse or forward primers were used in the reverse transcription step [13,14], using TaqMan Reverse Transcription Reagent kit (Applied Biosystems, Foster City, CA, USA). cDNAs were treated with 1μL of RNase H (2U/ μL) for 20’ at 37°C, to remove remaining viral RNAs, and then amplified using the RealStar Filovirus Screen RT-PCR Kit 1.0, with a modified thermal profile, which omitted the reverse transcription step. The standard curve of the RealStar Filovirus Screen RT-PCR Kit 1.0 has been used to determine the concentration of the virus in the different clinical specimens. In our experimental conditions, the range of the curve is from 10^9^ copies/mL down to 10^2^ copies/mL.

### Ethic statement

The Institutional Ethic Board (IEB) of the National Institute for Infectious Diseases “*L*. *Spallanzani*” approved the use of residual clinical samples for research purposes. The patient signed a written informed consent for any single procedure or treatment performed, after thoroughly explanation of reasonably anticipated benefits and potential hazards of intervention, and for the publication of the case.

All participants to the study are adults.
